# Oxidized and electronegative low-density lipoprotein as potential biomarkers of cardiovascular risk in obese adolescents

**DOI:** 10.6061/clinics/2018/e189

**Published:** 2018-10-05

**Authors:** Maria Camila Pruper de Freitas, Diana Gabriela Estevez Fernandez, Danielle Cohen, Antônio Martins Figueiredo-Neto, Raul Cavalcante Maranhão, Nágila Raquel Teixeira Damasceno

**Affiliations:** I Departamento de Nutricao, Faculdade de Saude Publica da Universidade de Sao Paulo, Sao Paulo, SP, BR; II Programa de Interunidades em Nutricao Humana Aplicada, Faculdade de Ciencias Farmaceuticas da Universidade de Sao Paulo, Sao Paulo, SP, BR; III Instituto de Fisica da Universidade de Sao Paulo, Sao Paulo, SP, BR; IV Laboratorio de Metabolismo de Lipides do Instituto do Coracao, Hospital das Clinicas, Faculdade de Medicina da Universidade de Sao Paulo, SP, BR; V Departamento de Analises Clinicas, Faculdade de Ciencias Farmaceuticas da Universidade de Sao Paulo, Sao Paulo, SP, BR

**Keywords:** Obesity, Adolescent, Lipoproteins, Low-density Lipoprotein

## Abstract

**OBJECTIVES:**

To evaluate biomarkers associated with early cardiometabolic risk in obese adolescents.

**METHODS:**

This cross-sectional study included 137 adolescents of both sexes aged 10 to 19 years divided into a normal weight group (NW) (n=69) and an obese group (OB) (n=68).

**RESULTS:**

As expected, obesity showed positive associations with homeostatic model assessment for insulin resistance (HOMA-IR), triacylglycerol, insulin, plasma levels of non-esterified fatty acids, and cholesterol ester transfer protein activity and negative associations with plasma antioxidant levels. Plasma oxidized low-density lipoprotein (oxLDL) and electronegative low-density lipoprotein [LDL(-)] levels were significantly higher in the OB group. Higher tertiles of oxLDL were associated with increased values of body mass index; waist circumference; fatty mass percentage (%FM); and the atherogenic lipids non-high-density-lipoprotein cholesterol (non-HDL-C), low-density lipoprotein cholesterol (LDL-C), apolipoprotein B and triacylglycerol. Higher tertiles of LDL(-) were robustly associated with body mass index and waist circumference. Logistic regression models (odds ratios) confirmed that increased values of lipids and apolipoprotein B were associated with increased risk of oxLDL. For LDL(-), these associations were not significant, suggesting that another mechanism is involved in generating this particle in obese adolescents.

**CONCLUSIONS:**

Obese adolescents showed increased plasma LDL(-) and oxLDL, and obese girls had more LDL(-) than obese boys. Therefore, oxLDL is strongly and independently associated with classical cardiovascular risk factors, while increased levels of LDL(-) were influenced by body mass index, waist circumference and demographic parameters in obese adolescents.

## INTRODUCTION

The increasing incidence of overweight children and adolescents around the world shows that these groups are more vulnerable to developing obesity 1,2. Similar to other countries, the prevalence of overweight adolescents in Brazil increased from 3.7% in 1975 to 21.7% in 2009 in boys and from 7.6% to 19% in girls. Currently, the rates of obesity in the adolescent population (10 to 19 years of age) are 6% for boys and 4% for girls 3.

In the general population, obesity is characterized by the excessive storage of body fat and is associated with an increased risk of developing chronic diseases, such as type 2 diabetes, high blood pressure, some types of cancer and cardiovascular diseases 1.

Obesity in childhood triples the risk of high low-density lipoprotein cholesterol (LDL-C), hypertriglyceridemia and hyperinsulinemia, and almost 60% of overweight adolescents have at least one cardiovascular risk factor 4.

In addition to the negative impact of obesity on the lipid profile, some studies in children have described an association between excess weight and oxidized low-density lipoprotein (oxLDL) 5. This modified LDL represents a lipoprotein subfraction directly involved in monocyte recruitment, foam cell formation and excessive accumulation of lipids in the artery wall, contributing to the development of atherosclerosis 6. Regarding the negative effects of oxidative changes in LDL, Avogaro et al. 7 identified an *in vivo* subfraction with increased negative charge, reduced antioxidant content and high-oxidized lipids, termed electronegative low-density lipoprotein [LDL(-)]. Despite the oxidative characteristics of LDL(-), other non-oxidative pathways are related to the generation of this particle, such as enrichment of non-esterified fatty acids (NEFAs), apolipoprotein CIII (Apo CIII), phospholipase A_2_ associated with lipoproteins (Lp-PLA_2_), glycation reactions and crosslinks with hemoglobin 8.

Because obesity is a growing problem in modern society and adolescence represents a susceptible period in which obesity can induce many pathways that negatively impact cardiovascular health, the primary aim of this study was to evaluate the potential associations of plasma oxLDL and LDL(-) with anthropometric and biochemical parameters in adolescents. We also evaluated their potential as biomarkers to identify cardiometabolic risk in obese adolescents.

## METHODS

### Study design and subjects

This cross-sectional study was conducted in adolescents between 10 and 19 years of age attending public schools in the west zone of Sao Paulo city, Brazil. For the present study, 137 individuals were enrolled. Underweight, pregnant, or lactating adolescents and those with chronic diseases or using drugs were not included. The study was approved by the Ethics Committee in Research (n° 1126/11), and information was obtained after obtaining informed consent.

After a nutritional evaluation, as proposed by Cole et al. 9, adolescents were divided into a normal weight group (NW) (n=69) and an obese group (OB) (n=68).

### Demographic and clinical data

All demographic and clinical data were collected through standardized surveys. Sexual maturation was analyzed by self-assessment, as proposed by Marshall 10 and Marshall and Tanner 11.

### Anthropometric parameters

Weight was evaluated using a Control II^®^ electronic digital balance (Plenna, Sao Paulo, Brazil) with a maximum capacity of 150 kg and an accuracy of 100 g; height was monitored with an Exact Height^®^ portable stadiometer (TBW, Sao Paulo, Brazil) with a limit of 2.1 m and an accuracy of 1 mm. From these data, body mass index (BMI) was calculated [weight (kg)/height (m^2^)] and classified according to Cole et al. 14, with considerations for sex and age. Waist circumference (WC) was assessed using inelastic tape with 1 mm precision (TBW, Sao Paulo, Brazil). The free-fat mass percentage (%FFM) and fat mass percentage (%FM) were measured by bioelectrical impedance (BIA) using the Biodynamics^®^ tetrapolar instrument, Model 450 (TBW, Sao Paulo, Brazil), through which a current of 800 μA and a frequency of 50 kHz were applied. These measures were performed in duplicate by trained professionals.

### Measurement of biochemical parameters

Blood samples were collected in vacutainer tubes containing ethylene diamine (EDTA, 1.0 mg/mL) (BD, Juiz de Fora, MG, Brazil) after 12h of fasting. The following protease inhibitors were added to the samples: aprotinin (10 µg/mL), benzamidine (10 µM), phenylmethylsulfonyl fluoride (PMSF, 5 µM) and the antioxidant butylated hydroxytoluene (BHT, 100 µM). Plasma and serum were separated by centrifugation (3000 rpm, 10 min, 4°C). The samples were stored at -80°C until analysis. Total cholesterol (TC), high-density lipoprotein cholesterol (HDL-C), and triacylglycerol (TAG) were determined by standard methods (Labtest Diagnóstica, MG, Brazil). The LDL-C levels were calculated using the Friedewald equation 12. Apolipoproteins AI (Apo AI) and B (Apo B) were evaluated with standard kits (RANDOX Co, Antrim, United Kingdom). Glucose (Glicose PAP Liquiform, Labtest, MG, Brazil) and insulin (Human Insulin-Specific RIA Kit, Linco Research, St Charles, MO, USA) were analyzed following the manufacturer's instructions. The homeostasis model assessment for insulin resistance index (HOMA-IR) was calculated using the following equation: HOMA-IR = (fasting insulin level (μU/mL) × fasing glucose (mmol/L)/22.5), as proposed by Matthews et al. 13. The enzymatic colorimetric assay HR Series NEFA-HR (2)^®^ (WAKO Diagnostics, Texas, USA) was used to determine non-esterified fatty acid concentrations (NEFAs). To measure the activity of cholesterol ester transfer protein (CETP), the standard CETP Activity Assay Kit (BioVision Research Products^®^, CA, USA) was used. All biochemical analyses were performed in duplicate, and the coefficient of variability was below 15%.

### OxLDL and LDL(-) analyses

OxLDL was detected by ELISA using a commercial kit (Mercodia AB, Uppsala, Sweden) according to the manufacturer's instructions.

LDL(-) was detected by sandwich ELISA following a protocol standardized by our research group 14. First, the plates (Costar^®^, model 3690, Corning, USA) were coated with monoclonal anti-LDL(-) (MAb-1A3) (0.5 µg/mL, 50 μl/well) diluted in carbonate-bicarbonate buffer (0.25 M, pH 9.6), and the plates were incubated overnight at 4°C. Free sites were then blocked with skim milk (Molico, Nestlé^®^, São Paulo, Brazil) diluted to 5% in phosphate-buffered saline 0.01 mol/L (PBS-Tween 0.01%, pH 7.4) and were incubated at 37°C for 2h. Then, the plates were washed four times with PBS-Tween (0.05%). Later, 50 µL/well of plasma diluted 1:1600 in PBS-Tween (0.01%) was added, and the plates were incubated for 2h at 37°C. Next, the plates were washed as previously described, and 50 µL/well of biotinylated monoclonal anti-LDL(-) (Mab 2C7) (0.12 µg/mL, 50 µL/well) was added. The plates were incubated at 37°C for 1h and were then washed as described above. After this step, 50 µL/well of streptavidin-peroxidase (1:60,000) diluted in PBS-Tween (0.01%) and 1% milk were added. The plates were incubated for 1h at 37°C and washed again, as previously described. The color reaction was developed by adding 3,3′,5,5′-tetramethylbenzidine (TMB) substrate comprising citrate-phosphate buffer (0.1 M, pH 4.2) and H_2_O_2_ (30%) (250/12/10, µL/mL/µL). The plates were incubated for 10 min at 37°C. The reaction was blocked with 50 μL/well H_2_SO_4_ (2 M), and the absorbance was monitored at 450 nm. The results were expressed as the mean absorbance minus the background sample and were then applied to the standard curve, multiplied by the corresponding titer, and expressed in U/L. The standard curve was calibrated with LDL(-) obtained from a plasma pool.

### Plasma antioxidant measurements

Plasma antioxidants (α-tocopherol, retinol and β-carotene) were analyzed by high-pressure liquid chromatography at the Vitae Clinics Laboratory. Antioxidants were extracted from plasma using commercial kits (Chromsystems, Munchen, Germany). All analyses were performed in duplicate, and antioxidant levels were determined using curves generated with external standards with multiple levels. The efficacy of the method was monitored using an internal standard.

### Statistical analysis

The statistical analysis was performed using SPSS 20.0 (Statistical Package for the Social Sciences) 15. The Kolmogorov-Smirnov test was used to evaluate the normal distribution of variables (*p*>0.05). The results are presented as the means and standard deviation (SD) or medians and interquartile range (p25 and p75). Comparisons between the NW and OB groups were evaluated using Student's t-test or the Mann-Whitney U-test. Comparison between tertiles of oxLDL and LDL(-) and other variables were tested by analysis of variance (one-way ANOVA) and Linear Trend Analysis, and the post-hoc Bonferroni test for multiple comparison adjustment was applied. To assess the hypothesis that plasma lipids can improve the probability of changes in oxLDL and LDL(-) in adolescents, logistic regression models were tested after multiple adjustments (age, sex, sexual maturation, BMI and WC). In the logistic regression models, the data were stratified into quartiles, and the 75^th^ percentile of oxLDL, LDL(-), Apo AI and Apo B was accepted as a cutoff point. For other lipid parameters, cutoff points were established according to previous guidelines: TC=150 mg/dL; LDL-C=100 mg/dL; HDL-C=45 mg/dL; TAG=100 mg/dL 16. For all analyses, the significance level adopted was *p*<0.05.

## RESULTS

**Figure 1:**
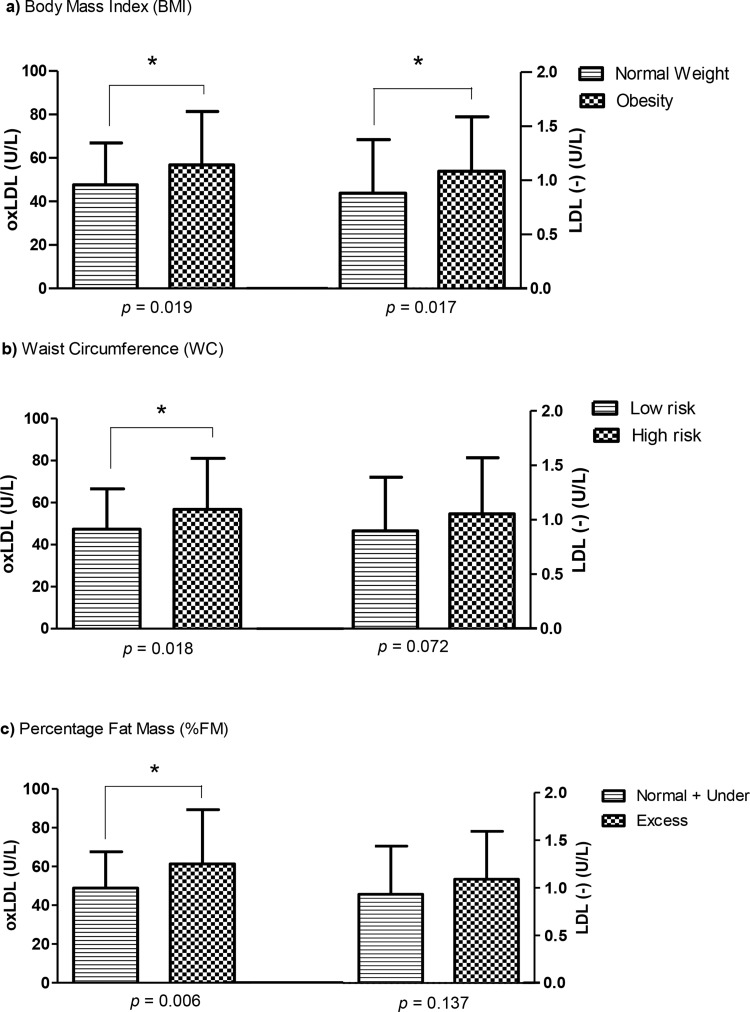
Oxidized LDL (oxLDL) and Electronegative LDL [LDL(-)] divided according to body mass index (BMI), waist circumference (WC), and fat mass (%FM). The data are shown as the means (standard deviation). *Student's t-test*. *Statistical significance *p*<0.05.

The NW and OB groups showed similar distributions of sex, age and sexual maturation, with higher frequencies of girls in both groups (NW: 66.67%; OB: 63.24%). More than 94% of the total sample was in the pubertal stage, and FM and WC data confirmed the nutritional status of adolescents classified according to BMI. As expected, obesity was associated with higher plasma levels of TAG, insulin, HOMA-IR, NEFAs, and CETP and lower plasma levels of HDL-C and Apo AI in the total samples. After sex stratification, a reduced value of HDL-C in obese girls was directly related to increased levels of CETP and NEFAs. In addition, the plasma Apo AI level was lower in both obese groups. In the OB group, plasma β-carotene and α-tocopherol levels were lower than in the NW group. Reduced antioxidant levels in the OB group were related to high contents of oxLDL and LDL(-). In girls, but not in boys, obesity was associated with higher values of LDL(-) (*p*=0.029) (Table 1). Plasma LDL(-) and oxLDL were significantly higher in the OB group than in the NW group. This profile was confirmed by higher values of oxLDL in adolescents with increased values of BMI, WC and FM. LDL(-) was significantly higher in obese adolescents (Figure 1).

Table 2 describes the linear trends for oxLDL and LDL(-), as well as anthropometric and lipid parameters. Higher tertiles of oxLDL were associated with increased values of BMI, WC and FM. Similar profiles were observed for non-HDL, LDL-C, TAG and Apo B. In contrast, higher tertiles of LDL(-) were related to higher BMI and WC values. This profile was confirmed by analysis of variance. Similarly, neither oxLDL nor LDL(-) were correlated (r=0.068, *p*=0.429) after dividing the adolescents by BMI (NW group: r=-0.012, *p*=0.919; OB group: r=0.061, *p*=0.621) and sex (girls: r=0.023, *p*=0.827; boys: r=0.139, *p*=0.347). Because some of the obese adolescents had normal lipid parameter values, we tested logistic regression models based on cutoff points for dyslipidemia. The odds ratios confirmed that none of the tested lipid variables showed significant improvements in LDL(-), except for Apo AI (AOR=2.980, *p*=0.028). In contrast, adolescents with higher values of TC, LDL-C, Apo B and TAG showed a three-fold higher probability of having oxLDL, while higher values of HDL-C and Apo AI were associated with a decrease in this particle, albeit not a significant one. These associations were independent of adjustments for age, sex, sexual maturation, body mass index and waist circumference (Table 3).

## DISCUSSION

The results presented in this study confirm that modifications in LDL are strongly influenced by different pathways in obese adolescents. The negative impact of obesity on cardiovascular diseases has been the focus of many studies 1; however, increasing rates of obesity in children and adolescents have stimulated new approaches 3. Similar to the well-established findings in obese adults, obesity in adolescents promotes metabolic abnormalities, such as dyslipidemia, hypertension, glucose intolerance, and type 2 diabetes 17,18. Our results show increased values of glucose (insulin and HOMA-IR) and lipid (TAG, NEFAs and CETP) parameters, which contribute to modifications of LDL. Obesity, particularly abdominal obesity, induces oxidative stress by increasing visceral fat deposition and NEFA release 19. Elevated NEFA levels promote hepatic TAG synthesis and, consequently, increase CETP activity. This protein modulates cholesterol ester transfer to apolipoproteins rich in Apo B, such as very-low-density lipoprotein (VLDL) and LDL, and it promotes HDL enrichment with TAG. In this process, hepatic lipase activity is stimulated, generating small, dense LDL, which is more prone to oxidation 20. According to Mello et al., at least 60% of small, dense LDL is represented by LDL(-) 21. Our results show that obese adolescents have increased values of oxLDL and LDL(-) in addition to unbalanced glucose and lipid parameters. Previously, Alves et al. showed that oxLDL was correlated with increased BMI, TAG, and LDL-C and decreased HDL-C 22. This set of features has also been described in other studies with obese adults and adolescents 23-25. Similarly, an evaluation of Latin adolescents by Ryder revealed that high oxLDL was associated with abdominal adiposity, increased insulin sensitivity, and TAG and decreased HDL-C levels 26. In our study, only oxLDL was associated with lipid risk factors. In contrast, LDL(-) did not show an association with the majority of biomarkers, except for Apo AI, suggesting that additional pathways link this marker to obesity. These differences can be partially explained by the fact that other non-oxidative pathways are involved in LDL(-) synthesis, such as enrichment with NEFAs, Apo CIII, Lp-PLA_2_, as well as glycation reactions and crosslinking with hemoglobin 8. Furthermore, LDL(-) is a LDL subfraction that shows increased levels of Apo CIII (lipase inhibitor), which regulates lipoprotein lipase in association with Apo CII (lipase activator), both of which contribute to TAG homeostasis and the generation of small, dense LDL 27.

Regarding the common oxidative basis linking oxLDL and LDL(-), we evaluated antioxidant vitamins in adolescents. Our results showed lower α-tocopherol and β-carotene levels in obese adolescents. Previously, Neuhouser et al. showed that obesity was related to low bioavailability of α-tocopherol and was directly related to the oxidative susceptibility of LDL 28.

In our study, a slight influence of sex on CETP and HDL-C was observed in obese girls, suggesting a potentially higher cardiovascular risk in this group. Based on the connection between CETP, HDL-C, and small, dense LDL, obese girls in our study showed increased LDL(-) values compared with girls of normal weight. Recently, Rao et al. used an elegant twin cohort design to show that changes in oxidized lipoproteins and Lp(a), an important carrier of oxidized phospholipids, were strongly mediated by heritability 29. That study adds a new mechanistic view to explain the differences in the risk of cardiovascular diseases by sex (men versus women) and hormonal status (premenopausal versus postmenopausal) that have been classically described in the literature 30. Hormonal and genetic parameters were not monitored in our study; however, previous scientific evidence has confirmed that both parameters influence lipid metabolism and LDL modifications (oxidative or not) 31. According to Lee et al., during puberty, increased estrogen levels prevent the oxidative DNA damage and endothelial senescence induced by LDL(-) 32. Our study was not able to confirm that finding; however, more than 94% of adolescents were in the pubertal stage, and both groups showed similar distributions between girls and boys. Nonetheless, obese girls had a worsened cardiovascular profile, indicating that other mechanisms can influence lipid metabolism and modifications of LDL during puberty, such as a higher percentage of fat mass, a common feature in females. To the best of our knowledge, our results are the first to provide evidence for the complementary role of LDL(-) and oxLDL in monitoring the risk of cardiovascular diseases in obese adolescents, reinforcing their potential as biomarkers. LDL(-) and oxLDL were not correlated in the total sample and were split by sex or obesity. Similarly, linear trend analysis and logistic regression models confirmed that although obese adolescents had more LDL(-), the variability was not explained by lipid parameters monitored in this study, except for a significant linear trend with BMI and WC. This possibility was shown in a recent review performed by Ivanova et al., which proposed LDL(-) as an independent cardiovascular risk factor 33. In contrast, Hsu et al. identified associations between LDL(-) and multiple cardiovascular risks, suggesting that this particle can be used as a novel cardiovascular risk factor 34. Several authors have described oxLDL and LDL(-) as pro-inflammatory and pro-atherogenic particles and have shown different features of these modified LDL 6,35. Others have found associations between obesity and oxLDL levels in adult populations 36,37 as well as in children and adolescents 5,35,38. Thus, a large-scale cohort study should be performed to establish oxLDL and LDL(-) as cardiovascular biomarkers or risk factors for cardiometabolic risk in adolescents.

Combined, these cardiovascular risk factors in association with modified LDL show a worsened profile in obese individuals, possibly contributing to early atherosclerosis processes, as proposed previously 39,40. Although clinical events rarely occur before the age of 60 years, monitoring classical risk factors and modified LDL in obese adolescents using intermediate outcomes, such as carotid intima-media thickness, could improve the prevention of cardiovascular diseases.

Significant odds ratios observed in our study contribute to ample research surrounding new biomarkers, cardiovascular risk factors in adolescence, and their impact in the causality of heart disease in adults. Our results confirm that modified LDL is part of the pathophysiology of obesity, and it is associated with other biomarkers in obese adolescents. Using adjusted logistic regression models, we showed that oxLDL was independently associated with multiple classical lipid markers; however, we highlight some pitfalls and limitations, including the function of different populations, life stage, sample size, analytical methods, and statistical approaches, as described in an elegant review by Brotman and Lauer (2005) 41.

In summary, cardiometabolic risk factors in obese adolescents may influence the modification of LDL particles in addition to the imbalance of glucose homeostasis and lipid metabolism. Obese adolescents show increased plasma LDL(-) and oxLDL levels, and obese girls have more LDL(-) than obese boys. Therefore, oxLDL was independently associated with multiple lipid markers, while increased values of LDL(-) were influenced by sex, age, sexual maturation, BMI, and WC in obese adolescents.

## AUTHOR CONTRIBUTIONS

Freitas MC designed and wrote the manuscript. Fernandes DG reviewed the manuscript and provided important advice. Cohen D helped performing the biochemical analyses. Maranhão RC, Figueiredo-Neto AM and Damasceno NR helped with the study design and critically reviewed the manuscript. All authors read and approved the final version of the manuscript.

## Figures and Tables

**Table 1:** Anthropometric and biochemical parameters in normal weight (NW) and obese (OB) adolescents.

Variables	NW			OB			*p*-value#
	Girls (n=46)	Boys (n=23)	Total (n=69)	Girls (n=43)	Boys (n=25)	Total (n=68)	
Age (years)	14.2 (2.1)	13.5 (2.2)	14.6 (2.2)	15.4 (2.1)	14.2 (2.5)	13.7 (2.3)	0.815
BMI (kg/m^2^)	20.4 (1.9)	20.4 (2.1)	20.4 (1.9)	32.1 (6.1)*	31.9 (4.8)**	32.0 (5.6)	**<0.001**
Waist circumference (cm)	68.3 (5.5)	70.9 (5.0)	69.2 (5.5)	93.9 (10.9)*	100.3 (12.4)**	96.3 (11.8)	**<0.001**
Fat mass (%)	22.3 (6.0)	11.8 (6.7)	18.8 (8.0)	34.2 (6.2)*	29.1 (5.6)**	32.3 (6.6)	**<0.001**
Free fat mass (%)	70.2 (13.7)	88.2 (6.7)	76.2 (14.6)	64.4 (7.1)*	70.9 (5.9)**	66.8 (7.3)	**<0.001**
Total cholesterol (mg/dL)	149.1 (31.8)	136.3 (34.4)	144.8 (33.0)	139.7 (45.1)	142.6 (27.3)	140.8 (39.3)	0.517
HDL-cholesterol (mg/dL)	44.2 (15.0)	39.2 (10.8)	42.5 (33.5)	34.9 (11.3)*	35.2 (12.6)	35.0 (11.7)	**<0.001**
Non-HDL-cholesterol (mg/dL)	104.9 (34.4)	104.9 (34.4)	102.3 (34.9)	104.9 (48.8)	107.4 (29.3)	105.8 (42.4)	0.596
LDL-cholesterol (mg/dL)	89.8 (34.4)	82.5 (34)	87.4 (34.2)	87.1 (43.9)	87.4 (28.7)	87.2 (38.7)	0.976
Triacylglycerol (mg/dL)	75.4 (29.4)	73.0 (31.4)	74.6 (29.9)	89.0 (48.6)	100.3 (42.1)**	93.1 (46.3)	**0.006**
Apo B (mg/dL)	62.6 (9.8)	64.3 (11.8)	63.2 (10.4)	65.5 (18.6)	62.8 (13.7)	64.5 (16.9)	0.586
Apo AI (mg/dL)	123.8 (19.5)	115.5 (13.4)	121.0 (181.0)	113.6 (24.8)*	104.8 (15.6)**	110.0 (22.2)	**0.002**
Glucose (mg/dL)	78.0 (13.2)	82.2 (12.2)	79.4 (12.9)	81.6 (11.7)	85.1 (8.9)	82.9 (10.8)	0.09
Insulin (µU/mL)	16.5 (8.0)	13.3 (5.0)	15.4 (7.3)	27.6 (16.6)*	24.1 (11.7)**	26.3 (15.0)	**<0.001**
HOMA-IR	3.2 (1.7)	2.7 (1.1)	3.0 (1.5)	5.6 (3.5)*	5.1 (2.7)**	5.5 (3.2)	**<0.001**
NEFAs (Eq/L)	520.0 (189.8)	431.0 (213.6)	490.4 (201.0)	682.0 (463.1)*	583 (255.1)**	645.4 (400.1)	**0.004**
CETP (pmol/µL/h)	28.9 (26.1)	40.3 (42.7)	32.7 (32.7)	53.8 (40.2)*	58.4 (50.6)	55.5 (44.0)	**<0.001**
oxLDL (U/L)	48.0 (17.3)	46.3 (23)	47.4 (19.2)	56.5 (24.5)	56.4 (24.3)	56.5 (24.2)	**0.017**
LDL(-) (U/L)	0.9 (0.5)	0.8 (0.5)	0.9 (0.5)	1.2 (0.5)*	1.0 (0.5)	1.1 (0.5)	**0.019**
β-carotene (µmol/L)	0.5 ( 0.7)	0.3 (0.2)	0.4 (0.6)	0.2 (0.1)*	0.2 (0.2)**	0.2 (0.1)	**<0.001**
Retinol (µmol/L)	1.4 (0.5)	1.6 (0.7)	1.4 (0.6)	1.3 (0.6)	1.6 (0.5)	1.5 (0.6)	0.883
α-tocopherol (µmol/L)	16.6 (5.1)	16.9 (5)	16.7 (5.0)	13.7 (5.1)*	14.3 (5.7)	14.0 (5.3)	**0.002**

The data are presented as the means (Standard Deviation). *Student's t-test* and *Mann-Whitney U test*. *Differences between girls in the OB group *vs* girls in the NW group. **Differences between boys in the OB group *vs* boys in the NW group. #Differences between total NW *vs* OB groups. Significance level (*p*<0.05). NW: normal weight; OB: obesity; BMI: body mass index; HDL: high-density lipoprotein; LDL: low-density lipoprotein; Apo B: apolipoprotein B; Apo AI: apolipoprotein AI; HOMA-IR: homeostasis model assessment for insulin resistance; NEFAs: non-esterified fatty acids; CETP: cholesterol ester transfer protein; oxLDL: oxidized LDL; LDL(-): electronegative LDL. Significant *p* values (<0.05) are in bold.

**Table 2:** Analysis of variance (ANOVA) and linear trend between oxLDL and LDL(-) and anthropometric and lipids parameters.

oxLDL (U/L)	1° tertile (n=45)	2° tertile (n=46)	3° tertile (n=46)	*p* for ANOVA	*p* for trend
	31.2 (29.4; 33.1)	47.8 (46.3; 49.2)	76.3 (70.5; 82.1)		
**Anthropometric variables**					
BMI (kg/m^2^)	23.8 (22.2; 25.5)*	27.0 (24.6; 29.5)	27.6 (25.5; 29.6)*	**0.026**	**0.013**
WC (cm)	76.9 (73.0; 80.8)*	83.9 (78.2; 89.5)	87.0 (82.6; 91.5)*	**0.009**	**0.003**
FM (%)	22.5 (19.6; 25.5)*	26.6 (23.7; 29.4)	27.4 (24.5; 30.3)*	**0.026**	**0.007**
**Lipid variables**					
non-HDL-C (mg/dL)	89.5 (81.5; 97.5)*	98.6 (89.9; 107.4)	123.6 (109.3; 138.0)*	**<0.001**	**<0.001**
Apo AI (mg/dL)	121.2 (113.3; 129.1)	111.4 (106.6; 116.3)	114.8 (109.3; 120.3)	0.059	0.115
Apo B (mg/dL)	57.0 (53.4; 60.6)*	61.8 (59.2; 64.4)**	72.6 (68.0; 77.3)*,**	**<0.001**	**<0.001**
HDL-C (mg/dL)	40.5 (36.6; 44.4)	36.5 (33.0; 39.9)	39.4 (34.9; 43.9)	0.362	0.641
LDL-C (mg/dL)	74.8 (66.9; 82.7)*	83.4 (74.9; 91.9)**	103.4 (89.9; 116.9)*,**	**<0.001**	**<0.001**
TAG (mg/dL)	73.8 (65.8; 81.7)	76.2 (65.9; 86.5)	101.2 (86.6; 115.9)	**0.001**	**0.001**

LDL(-) (U/L)	1° tertile (n=45)	2° tertile (n=46)	3° tertile (n=46)	*p* for ANOVA	*p* for trend
	0.4 (0.3;0.4)	1.0 (1.0;1.1)	1.5 (1.5;1.6)		
**Anthropometric variables**					
BMI (kg/m^2^)	24.2 (22.3; 26.1)	27.1 (24.7; 29.4)	27.22 (25.3; 29.2)	0.072	**0.037**
WC (cm)	78.3 (73.6; 83.0)	84.7 (79.5; 89.9)	84.8 (80.3; 89.4)	**0.047**	**0.022**
FM (%)	22.5 (19.5; 25.5)	26.5 (23.7; 29.4)	27.4 (24.5; 30.3)	0.135	0.078
**Lipid variables**					
non-HDL-C (mg/dL)	108.2 (95.7; 120.7)	99.4 (88.8; 109.9)	104.7 (93.0; 116.3)	0.585	0.622
Apo AI (mg/dL)	116.0 (108.0; 124.1)	115.0 (110.4; 120.0)	116.3 (110.5; 122.0)	0.837	0.676
Apo B (mg/dL)	63.7 (59.5; 68.0)	64.0 (59.9; 68.2)	63.8 (59.6; 68.0)	0.998	0.962
HDL-C (mg/dL)	40.3 (36.2; 44.4)	38.8 (35.1; 42.5)	37.3 (33.1; 41.4)	0.525	0.180
LDL-C (mg/dL)	90.2 (77.8; 102.5)	84.3 (74.4; 94.1)	87.5 (77.1; 98.0)	0.747	0.661
TAG (mg/dL)	90.2 (76.4; 104.1)	75.6 (66.5; 84.6)	85.7 (73.6; 97.9)	0.264	0.699

The results are shown as the means (confidence interval - CI). Analysis of variance (one-way ANOVA) between tertiles and Bonferroni test for multiple comparisons adjustment. *Differences between 2**°** and 3**°** tertiles *vs* 1 tertile. **Differences between 2**°** tertile and 3**°** tertile. Models adjusted by age, sex, and sexual maturation. Significance *p*<0.05. BMI: body mass index; WC: waist circumference; FM: fatt mass; HDL-C: high-density lipoprotein cholesterol; Apo AI: apolipoprotein AI; Apo B: apolipoprotein B; LDL-C: low-density lipoprotein cholesterol; TAG: triacylglycerol. Significant *p* values (<0.05) are in bold.

**Table 3:** Multiple Logisitic Regression Models for impact of lipid profile in modified low-density lipoprotein: oxLDL and LDL(-).

Variables	oxLDL (*p*>75)		LDL(-) (*p*>75)	
	AOR	*p*	AOR	*p*
TC				
* <150 (mg/dL)*	ref.		ref.	
* ≥150 (mg/dL)*	4.334	**0.001**	0.773	0.559
LDL-C				
* <100 (mg/dL)*	ref.		ref.	
* ≥100 (mg/dL)*	3.990	**0.002**	0.591	0.278
HDL-C				
* <45(mg/dL)*	ref.		ref.	
* ≥45(mg/dL)*	0.669	0.408	0.727	0.524
TAG				
* <100 (mg/dL)*	ref.		ref.	
* ≥100 (mg/dL0)*	7.558	**<0.001**	0.799	0.642
Apo AI*				
* <p75*	ref.		ref.	
* ≥p75*	0.919	0.870	2.980	**0.028**
Apo B*				
* <p75*	ref.		ref.	
* ≥p75*	8.816	**<0.001**	0.730	0.537

n=137. The cutoff points were established according to V Brazilian Guidelines on Dyslipidemias and Prevention of Atherosclerosis 16. *For Apo AI and Apo B, the cutoff points adopted were percentile 75 (*p*75). AOR: Models adjusted by age, sex, sexual maturation, body mass index, and waist circumference. Abbreviations: TC: total cholesterol; LDL-C: low-density lipoprotein cholesterol; HDL-C: high-density lipoprotein cholesterol; TAG: triacylglycerol; APOAI: apolipoprotein AI; APOB: apolipoprotein B. Significant *p* values (<0.05) are in bold.
